# Association of physical activity and heart rate variability in people with overweight and obesity: A systematic review

**DOI:** 10.12688/f1000research.124707.1

**Published:** 2023-02-10

**Authors:** Mukesh Kumar Sinha, Vaishali K., Arun G. Maiya, Shivashankar K.N., Shashikiran U., Ravi Shankar N.

**Affiliations:** 1 Department of Physiotherapy, Manipal College of Health Professions, Manipal Academy of Higher Education, Manipal, Karnataka, 576104, India; 2 Department of Medicine, Kasturba Medical college, Manipal Academy of Higher Education, Manipal, Karnataka, 576104, India; 3 Department of Medicine, Dr. TMA Pai Hospital, Udupi, MMMC, Manipal Academy of Higher Education, Manipal, Karnataka, 576104, India; 4 Department of Biostatistics, Vallabhbhai Patel Chest Institute, University of Delhi, Delhi, Delhi, India

**Keywords:** exercise, cardiac autonomic function, cardiorespiratory endurance, aerobic exercise, resistance exercise

## Abstract

**Background: **Obesity is a major public health issue globally which is intrinsically linked to reduced heart rate variability (HRV). Physical inactivity and reduced resting HRV are linked to an increased risk of coronary heart disease, while athletes have a greater HRV. However, the exact correlation between physical activity and HRV remains uncertain. This systematic review aims to collect, report, and critically assess the current scientific literature about the association between physical activity and HRV in individuals with higher weight and obesity.

**Methods:** A systematic search was carried out in electronic databases (Medline/PubMed, SCOPUS and CINAHL Plus) to retrieve studies that evaluated the relationship between physical activity and HRV in individuals with higher weight and obesity. Case-control, longitudinal/cohort, cross-sectional and observational studies were included. Using a critical narrative approach, information about the HRV, and physical activity was extracted and synthesized. The study was registered in PROSPERO: CRD42020208018 on October 9 2020.

**Results: **After removing duplicates, 980 title/abstract records were checked for eligibility, and 12 papers were finally included in the narrative synthesis. The included studies contained physical activity as well as HRV in adults with higher weight or obesity with or without comorbidities. A negative relationship between moderate to vigorous physical activity and HRV indices had been found in two studies. There was also a negative relationship between sedentary time and HF (p = 0.049) and LF/HF (p = 0.036), as well as a positive relationship between sedentary time and LF (p = 0.014). Also dose-response association was found between vigorous exercise and higher SDNN, LF power, and HF power in one of the studies.

**Conclusions:** This systematic review revealed a wide range of responses to physical activity and HRV; however, the current evidence uses a variety of approaches to objectively assess physical activity and measure HRV with different equipment.

## Abbreviations

HRV: Time-domain variables of Heart rate variability

SDNN: Standard deviation of NN intervals (ms)

RMSSD: Root mean square of successive RR interval differences (ms)

pNN50: Percentage of successive RR intervals that differ by more than 50 ms (%). Frequency domain variables of Heart rate variability

VLF: Absolute power of the very-low-frequency band (0.0033–0.04 Hz)– milli-second square

LF: Power in the low-frequency range (0.04–0.15 Hz)

HF: Power in the high-frequency range (0.15–0.4 Hz)

LF/HF ratio: Ratio of LF [ms ^2^]/HF [ms ^2^], Ln, the natural logarithm of the absolute value in ms2

BMI: Body mass index

WC: Waist circumference

ST: Sedentary time

BF%: Body fat %

GPCS: Global physical capability score

PA: Physical activity

LPA: Light-intensity PA

VPA: Vigorous-intensity PA

VVPA: Mean daily very vigorous physical activity

WHR: Waist-hip ratio

HR: Heart rate

MVPA: Moderate-to-vigorous physical activity

CRF: Cardiorespiratory fitness

MBQOA: The Modified Baecke Questionnaire for Older Adults

PAEE: Physical activity energy expenditure

IPAQ: International Physical Activity Questionnaire

## Introduction

Having higher weight or obesity is characterized by abnormal or excessive fat accumulation that poses a health concern leading to chronic medical issues and a greater risk of disability. ^1^^,^^2^ Obesity affects about 40% of young adults aged 20-39 years, 44.8% of middle-aged adults (aged 40-59 years), and 42.8 % of people aged ≥ 60 years globally. ^2^ The World Health Organization estimated the global economic impact of obesity to be around 2.8% of the world’s gross domestic product. However, despite its widespread prevalence, and the accompanying cost, disease burden, and complications, obesity is often ignored as a disease. ^3^ In the low-and-middle-income nations, uncontrolled urbanization and a shift in eating patterns from traditional to western-style diets are leading to an alarming rise in the incidence of obesity. It is reported that a curvilinear relationship between relative weight and mortality begins after the age of 18 years. ^1^

Several diseases like diabetes, hypertension, cancer, stroke, osteoarthritis, liver, renal disease, sleep apnea, and depression are linked to obesity, and the association increases with age and the presence of comorbidities. ^4^^–^^21^ Another notable dysfunction associated with obesity is the disruption of cardiac autonomic function leading to alterations in the normal parasympathetic and sympathetic regulation, which can be detected using heart rate variability (HRV). ^22^^–^^25^ HRV is a non-invasive tool for evaluating autonomic function by measuring beat-to-beat differences in R-R intervals. ^26^ It is known that low HRV is an independent predictor of cardiovascular mortality and sudden cardiac death, is related to higher skinfold thickness, higher body mass index (BMI), higher body fat percentages, and lower levels of physical activity. ^27^^–^^29^ Furthermore, reduced physical activity and cardiorespiratory fitness are also potential risk factors contributing to cardiovascular diseases ^30^ which are worsened by higher associated adiposity indices. ^31^ Research shows that increased epicardial fat thickness is related to a lack of physical activity, impacting the autonomic nervous system. ^32^^–^^34^ Despite these facts, the relationship between physical activity and the autonomic cardiac function as measured by HRV has not been explored well. Accordingly, this systematic review aimed to collate, review and critically assess current scientific and clinical knowledge on the relationship between physical activity and HRV in adults with either higher weight or obesity.

## Methods

The study was registered in PROSPERO: CRD42020208018 on October 9 2020.

### Search strategy

Search strategies were developed related to physical activity and HRV with the use of boolean operators “AND,” and “OR” to allow logical interconnections between concepts. Synonyms or Medical Topic Heading (MeSH) keywords and the title and abstract text for each of the terms were searched. Multiple databases, Medline, SCOPUS, Cumulative Index to Nursing, and Allied Health Library (CINAHL Plus) [Ebscohost], were searched from their earliest record to January 2021. Before adaptation for the other databases, the search strategy was initially built in Medline. For any additional studies, the reference list entries of the included studies and systematic reviews were inspected. We used the following keywords in the quest–exercise, physical activity, incidental physical activity, aerobic fitness, fitness, habitual physical activity, obese, overweight, healthy people, older adults, cardiac autonomic function, autonomic nervous system, heart rate variability, autonomic function, sympathetic function, and parasympathetic function. Table 1 presents the search strategy in detail.

**Table 1.** Search quest.

**Physical activity related search term:** (((((((((((((((exercise) OR (exercise [MeSH Terms])) OR (exercise [Title/Abstract])) OR (exercise [Title])) OR (physical activity)) OR (physical activity [Title/Abstract])) OR (physical activity [Title])) OR (incidental physical activity)) OR (incidental physical activity [Title/Abstract])) OR (incidental physical activity [Title])) OR (aerobic fitness)) OR (aerobic fitness [Title/Abstract])) OR (aerobic fitness [Title])) OR (fitness)) OR (fitness [Title/Abstract])) OR (fitness [Title])
AND
**Population:** (((((((((((obesity [Title]) OR (obesity)) OR (obesity [Title/Abstract])) OR (obesity [MeSH Terms])) OR (overweight [Title])) OR (overweight)) OR (overweight [Title/Abstract])) OR (overweight [MeSH Terms])) OR (healthy people [Title/Abstract])) OR (healthy people [Title])) OR (older adults [Title])) OR (older adults [Title/Abstract])
AND
**Heart rate variability related search term:**
((((((((((((((cardiac autonomic function [Title/Abstract]) OR (cardiac autonomic function [Title])) OR (autonomic function [Title])) OR (autonomic function [Title/Abstract])) OR (autonomic nervous system [Title/Abstract])) OR (autonomic nervous system [Title])) OR (autonomic nervous system [MeSH Terms])) OR (autonomic nervous system)) OR (heart rate variability)) OR (heart rate variability [Title/Abstract])) OR (heart rate variability [Title])) OR (sympathetic function [Title])) OR (sympathetic function [Title/Abstract])) OR (parasympathetic function [Title/Abstract])) OR (parasympathetic function [Title])

### Eligibility criteria

Only case-control, longitudinal/cohort, cross-sectional, and observational studies about physical activity (occupational, transportation, and leisure) and HRV in people with higher weight and obesity with or without any comorbidity were included in this review. A reported mean or median BMI value of ≥ 25 kg/m ^2^ was also considered for inclusion. The physical activity assessment in the studies could be either subjectively or objectively evaluated. The HRV considered for inclusion was either normalized unit or log-transformed data of time domain or in frequency domain measures; all time-domain measures were reported in ms and frequency domain measures in ms ^2^. Studies that failed to examine the results of interest by using only healthy subjects or not including the overweight and obese group were excluded from the analysis.

### Study selection

MKS screened all study titles after removing duplicates. Then, MKS and VK independently assessed all relevant titles and abstracts for eligibility, and the full texts of all potentially qualifying papers for independent review were retrieved. Any disputes were addressed by dialogue with the third reviewer, AGM. Articles considered suitable were further processed for data extraction and quality assessment.

### Data extraction

The following data were extracted from each qualifying article: study design, study setting, funding source, ethical approval, study population characteristics (age, gender, BMI, body fat percentage), inclusion criteria, obesity grading, sample size, objectives, outcome measures, and compiled in a data-recording table. Initially, MKS and VK piloted the data extraction and qualitative evaluation methods on three papers. MKS conducted data extraction and it was checked by VK. Any conflicts were resolved by contacting the third reviewer, AGM, SK, SU and RS.

### Data analysis

A narrative synthesis of the studies was performed while accounting for the differences in the individual investigations regarding their design, findings, and objectives. A meta-analytical approach was not feasible in this analysis due to variations in the measurement of physical activity and HRV and the different study designs.

## Results

### Literature identified through the search strategy

**Figure 1.**
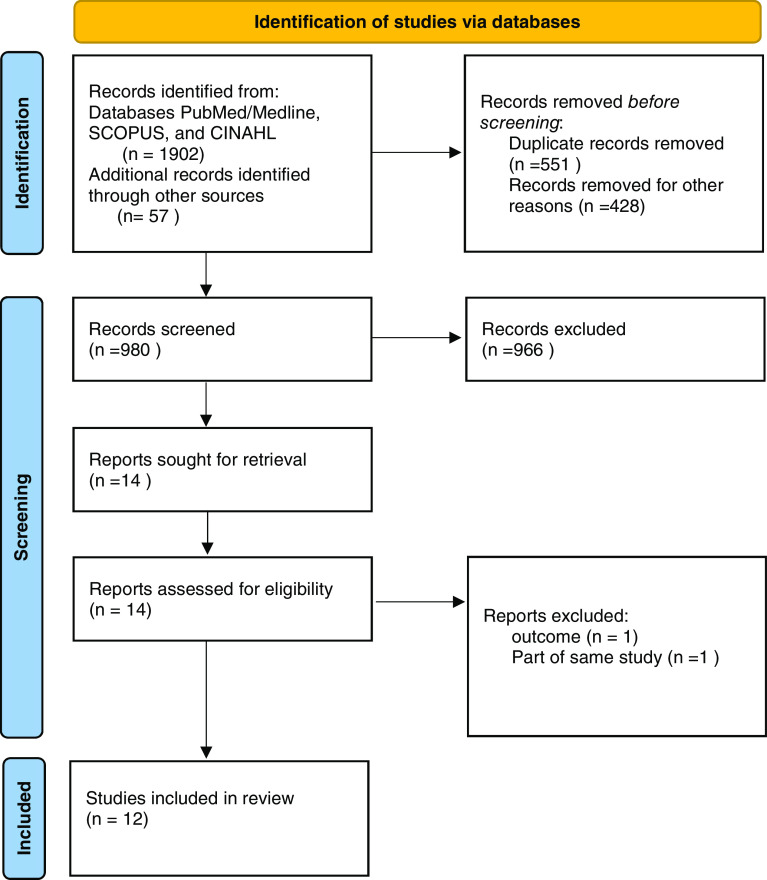
PRISMA Flow diagram.

We retrieved 1,902 published papers following a database search based on the keywords described above. After removing the duplicates, 980 records of titles/abstracts were screened for eligibility, of which, 12 studies were finally included in the narrative synthesis of this systematic review. A Preferred Reporting Items for Systematic Reviews and Meta-Analyses (PRISMA) flow diagram for the inclusion process is presented in Figure 1. A summary of the 12 studies is presented in Table 2. ^23^^,^^35^^–^^45^

**Table 2.** Characteristics of included studies.

Study	Study design	Participants	Methods	Results
**1**. **Oliveira *et al***: Journal of Obesity. 2020	**An Observational study (clipping from RCT)**	**Sample Size:** 64 patients with obesity. Participants: Obesity grade II (BMI ≥35 kg/m ^2^). Either gender and age of between 18 and 65 years.	**Anthropometric measurement**: BMI, WC **Physical activity:** Accelerometer ACTi Graph model wGT3X, and 24 h recall (R24H) **HRV measurement equipment, procedure, and reported domain** **Equipment**: V800 Polar, Finland. Kubios HRV Analysis software, version 2.2 **Procedure**: Room temperature was maintained at 22-24 ^o^C and relative humidity of 50-60%. On the day of measurement and 24 hours before, participants were advised not to drink alcohol, perform moderate or intense PA and avoid copious meals. **Reported domain**: Frequency domain measures of HRV- LF, HF, and LF/HF ratio	Negative association b/w: moderate to vigorous PA and the sympathetic component of HRV (p = 0.043). Negative association b/w Sedentary time with HF and LF/HF. Positive association observed b/w Sedentary time with LF. Waist circumference was negatively associated with HF (β = −0.685, p = 0.010).
**2. De Liao *et al***: PLOS ONE. 2017	**Cross-sectional study**	**Sample size:** 441 (231 males and 210 females). Participants: Older adults with obesity of either gender aged 50-80 years. BF% of the participants: >27% for men and >38% for women	**Anthropometric measurement**: (BMI, Body fat (BF)%). **Physical activity:** GCPS score **HRV measurement equipment, procedure, and reported domain** **Equipment:** ANS watch monitor (Taiwan Scientific Co., Taipei, Taiwan) **Procedure:** Participants were given rest for 10 min in a supine position. HRV analysis: Pulse cycle intervals instead. **Reported domain**: Time and frequency domain variables- SDNN, rMSSD, and HF	Moderate association was observed between - The GCPS with SDNN and HF in older men. similar findings were found in older women.
**3. Pope *et al***: Medicine & science in sports & exercise. 2020	**Cohort study**	**Sample size:** 1668 participants. men and women, age of 18-30 years. The majority of the participants were with higher weight and obesity - n=1157 Underweight - n=20 Normal weight - n=491	**Anthropometric measurement:** BMI **Physical activity:** ActiGraph 7164 (ActiGraph Corp.; Pensacola, FL) **HRV measurement equipment, procedure, and reported domain** **Equipment:** GE MC1200 (General Electric Inc., Boston, MA) **Procedure:** Resting ECG recording data at 500 Hz, with three 10-s ECG strips. **Reported domain:** Time-domain measure of HRV- SDNN and rMSSD	VPA was associated with SDNN and rMSSD and LPA was associated with rMSSD only.
**4. Tonello *et al***: Frontiers in physiology. 2016	**Correlation study**	**Sample Size:** 21 Young, healthy non-menopausal women with higher weight free from pathological conditions. Mean age = 34.5 ± 6.40 years. Mean BMI = 26.3 ± 4.1 kg/m ^2^. Mean WC = 79.7 ± 9.7.CM Mean BF% = 37.0 ± 4.7. Full-time service worker. Not involved in any structured exercise program	**Anthropometric measurement:** BMI, WC, body fat% **Physical activity:** Measured using accelerometer (GT1M, ActiGraph USA) **HRV measurement equipment, procedure, and reported domain** **Equipment:** HR monitor- RS800CX, Polar Electro Oy, Finland **Procedure:** Sampling rate- 1000Hz Software used for filter- (Polar ProTrainer ^®^ version 5.0, POLAR Electro Oy, Kuopio, Finland). Software used for HRV analysis- Kubios HRV v2.0, Kuopio University, Finland. Orthostatic and ambulatory HRV recording was performed **Reported domain:** Time-domain measures- SDNN and RMSSD, Frequency domain measures-LF and HF	In adult women with higher weight, incidental or non-exercise-based PA was associated with increased autonomic reactivation. Correlations were observed between: VPA and RMSSD (r = -0.449, p = 0.041), VPA and HF (r = -0.520, p = 0.016), VPA and SD1 (r = -0.463, p = 0.035), VPA +VVPA and RMSSD (r = -0.453, p = 0.039), VPA +VVPA and HF (r = -0.526, p = 0.014), VPA +VVPA and SD1 (r = -0.473, p = 0.030).
**5. Kaikkonen KM *et al***: Journal of Physical Activity and Health. 2014	**Cross-sectional study**	**Sample Size:** 107 adults with obesity (87 females and 20 males) Obese adults with a mean age of 44.5 years and median BMI of 35.7	**Anthropometric measurement:** BMI and WC **Physical activity:** Modified Paffenbarger questionnaire. Self-rated physical fitness assessment: Standardized 5-scale question with categories “very poor,” “fairly poor,” “satisfactory,” “fairly good,” and “very good.” **HRV measurement equipment, procedure, and reported domain** **Equipment:** Polar R-R recorder (Polar Electro Oy, Kempele, Finland) **Procedure:** Software used for analysis - Heart Signal Co, Kempele, Finland. The recording was carried out for 24 hours and HRV was computed from the entire recording. RR intervals were visually inspected to remove ectopic beats. **Reported domain:** Time-domain (SDNN), Frequency domain- LnHF, LnLF, LnVLF, LnULF, LF/HF ratio.	Lifetime physical activity explained 40% of the variance in SDNN in multivariate linear regression analyses. SDNN increased by 15.4 (P =.009) and 24 % of the variance in LnULF (P =.050) increased with each 1 group increase in the activity index.
**6. Rennie *et al***: American Journal of Epidemiology. 2003	**Cohort study (Data presented from the fifth** **phase of data collection)**	Whitehall II study of civil servants age between 45–68 years. **Sample Size:** 3,328 participants (994 women). Participant BMI included above and below 25. Waist-hip ratio: 0.793-0.941	**Anthropometric measurement:** BMI and WHR **Physical activity:** Physical activity questionnaire derived from Minnesota leisure-time activity questionnaire **HRV measurement equipment, procedure, and reported domain** **Equipment:** 12-lead electrocardiogram (Mingorec; Siemens, Munich, Germany) **Procedure:** Participants were made to rest supine for at least 5 minutes in a quiet room. Five minutes of beat-to-beat heart rate data were sampled at a frequency of 500 Hz. Software used for digital recording of R wave - (Kardiosis; Tepa, Inc., Ankara, Turkey) RR intervals were visually inspected to remove ectopic beats. **Reported domain:** Time-domain (SDNN), Frequency domain (LF and HF)	There was a clear dose-response relationship between vigorous activity and higher SDNN, low-frequency power, high-frequency power (p 0.05, p 0.01, and p 0.01, respectively) in men.
**7. Kiviniemi AM *et al***: Medicine & science in sports & exercise. 2017	**Prospective Northern Finland** **Birth Cohort 1966 (NFBC1966) study**	At the age of 46 years, 1383 men and 1761 women without cardiorespiratory diseases and diabetes underwent assessments of HRV. Nonsmoker: n (%) Men - 589 (43), women - 905 (51) * Ex-smoker: n (%) Men - 410 (30), women - 423 (24) Current smoker: n (%) Men - 384 (28), Women - 433 (25) .*P < 0.001 compared with men.	**Anthropometric measurement:** BMI, Body fat, %, and Waist-hip ratio **Physical activity:** PA was objectively measured with a wrist-worn Polar Active device (Polar Electro Oy, Kempele, Finland) **HRV measurement equipment, procedure, and reported domain** **Equipment:** HR monitor (RS800CX; Polar Electro Oy) was used to record R-R intervals (RRi). Standard lead II ECG (Cardiolife; Nihon Kohden, Tokyo, Japan), breathing frequency (MLT415/D, Nasal Temperature Probe; AD Instruments, Bella Vista, New South Wales, Australia), and blood pressure (BP) by finger photoplethysmography (Nexfin; BMEYE Medical Systems, Amsterdam, the Netherlands) were recorded with a sampling frequency of1000 Hz (Power Lab 8/35; AD Instruments). **Procedure:** The first 150 s of 3-min recording was used for analysis. Spontaneous breathing was allowed during the HRV recording. Artifacts and ectopic beats were removed and replaced by the local average (Hearts 1.2; University of Oulu, Oulu, Finland). Sequences with ≥10 consecutive beats of noise or ectopic beats were deleted before final analysis. The RRi series with ≥80% accepted data were considered for analysis. **Reported domain:** Time-domain RMSSD (ms)	• MVPA was independently associated with cardiac autonomic function in both men and women. • Body fat was independently associated with cardiac autonomic function in both men and women. • A negative association was found between MVPA and rMSSD when including CRF, MVPA, and Fat% in the same regression model. • Body Fat% was not significantly related to rMSSD.
**8. Föhr T *et al*:** BMC public health. 2016	**Cross-sectional study**	Finnish employees: (6863 men and 9412 women; Age - 18–65 years old; BMI: [18.5–40.0 kg/m ^2^; mean = 26.0 ± 4.1 kg/m ^2^].	**Anthropometric measurement:** BMI **Physical activity:** HRV-based assessment of PA. The amount and intensity of PA were determined using ambulatory beat-to-beat R-R interval data. **HRV measurement equipment, procedure, and reported domain** **Equipment:** Firstbeat Bodyguard device (Firstbeat Technologies Ltd., Jyväskylä, Finland). **Procedure:** Ambulatory ECG: continuous beat-to-beat R-R interval (ECG) recordings during their everyday life. R-R interval data were analyzed using Firstbeat Analysis Server software (Firstbeat Technologies Ltd, Jyväskylä, Finland. A 5-minute window was used to measure RMSSD. If a subject's measurement duration included two or more workdays, an average was used for analysis. **Reported domain:** RMSSD and the LF/HF ratio	For both men and women, the high PA group had the highest rMSSD (during waking hours and during sleep) and recovery index, and the lowest stress percentage and stress index. Both moderate and vigorous PA is found to be associated with higher HRV
**9. Buchheit M, *et al* ** . Medicine, and science in sports and exercise. 2005	**Observational study**	43 non-smoking, middle-aged adults without obesity. 23 women 20 men Age: 61.2 ±4.3 years old BMI: 25.6 ± 0.3 kg/m ^2^	**Anthropometric measurement:** BMI **Physical activity:** MBQOA Questionnaire. The total score reflects overall PAEE. Physical activity parameters were assessed by triaxial accelerometers (RT3, Stayhealthy, Monrovia, CA) **HRV measurement equipment, procedure, and reported domain** **Equipment:** ECG was continuously monitored using a Holter with a sampling frequency of 256 Hz (Ela Medical, Paris, France). Breathing frequency was monitored using Crystal Trace Piezo Respiration Sensor (Astro-Med EEG System, Grass Instruments, West Warwick, RI). **Procedure:** Recording Time: Between 10:00 a.m. and 12:00 p.m. Recording environment: air-conditioned room with ambient temperature maintained at 21°C. Following 30 mins of rest, subjects were asked to remain quietly supine for 10 mins without speaking or making any movements. HRV analyses were considered from the last 5 min of the 10-min controlled breathing period. **Reported domain:** mean of R–R intervals (mR–R), SDNN, RMSSD, LF, HF, and LF/HF ratio.	Habitual moderate PAEE is associated with higher vagal indexes of the heart in older adults.
**10. May *et al* ** . Journal of physiological anthropology. 2017	**Short report**	115 students Age: 23.1 ± 5.4 years old BMI: 25.4 ± 0.5 kg/m ^2^	**Anthropometric measurement:** BMI **Physical activity:** IPAQ **HRV measurement equipment, procedure, and reported domain Equipment:** Electrocardiographic recordings were collected using a BIOPAC MP-36 system (BIOPAC Systems Inc., Galeta, Ca). Software: BIOPAC Student Lab Pro software (BIOPAC Systems, Inc.) and values were natural log-transformed. **Procedure:** Recording Time: 0800-1030 hours Recording environment: Temperature-controlled room. Participants were seated and were instructed to breathe normally with their eyes closed. Following 5 mins of quiet rest, 5-min recordings were collected with a sample rate of 1000 samples/s. **Reported domain:** rMSSD, LF and HF	In regression analyses, hours of vigorous physical activity significantly predicted greater time domain and frequency domain indices of HRV.
**11. Soares-Miranda *et al*.** Circulation. 2014	**Prospective study (The Cardiovascular Health Study (CHS) design)**	5,201 ambulatories, non-institutionalized men and women ≥ 65 years of age were randomly selected and enrolled from Medicare eligibility lists in 4 US communities in 1989–990. BMI: 27 ± 5 kg/m ^2^	**Anthropometric measurement:** BMI **Physical activity:** modified Minnesota Leisure-Time Activities questionnaire. **HRV measurement equipment, procedure, and reported domain** **Equipment:** Long-term (e.g., 24-hour Holter) measures. Two-channel 24-hour Holter recordings (Del Mar Medical Systems, Irvine, California). HRV analysis was done in HRV Laboratory (GE Marquette Mars 8000 Holter analyzer, Milwaukee, Wisconsin). **Procedure:** Recording at baseline in 1989–90 and again in 1994–95. For cross-sectional analyses at baseline in 1989–90, 1,219 participants had 24-hour recordings for time-domain HRV and 1,150 for frequency-domain and nonlinear HRV. **Reported domain: SDNN,** rMSSD, SDNN Index, LF, HF, ULF, VLF, LF/HF ratio, SD12, Poincare Ratio, and DFA1	Following multivariable adjustment, the leisure-time activity was cross-sectionally related to specific indices including higher SDNN (p trend=0.001) and higher ULF (p trend<0.0001).
**12. Kluttig *et al* ** . BMC cardiovascular disorders. 2010	**Part of Prospective, population-based CARLA study (Cohort study)**	A cross-sectional data of 1671 participants. Men (n = 967), Women (n = 812) Age range - 45-83 years old BMI: Male: 28.5 ± 4.1 kg/m ^2^ Female: 28.5 ± 5.4 kg/m ^2^	**Anthropometric measurement:** BMI, WHR **Physical activity:** Baecke questionnaire **HRV measurement equipment, procedure, and reported domain** **Equipment:** Modular ECG Analysis System (MEANS) **Procedure:** The 20-min ECG was recorded after a resting period (in the supine position) of ≥20 min. Throughout the ECG, subjects were asked to breathe at 15 breaths/min (0.25 Hz) Time and frequency domain parameters of HRV for 5-min segments of the ECG according was used for the HRV analysis. **Reported domain:** SDNN, LF, HF, and LF/HF ratio	There was no consistent or statistically significant association of physical activity with HRV in either gender was observed.

### Quality assessment of included studies

The quality of the studies was determined by the quality index score. ^46^ For the present review, the score for the included studies ranged from 16 to 23; however, a few of the points were not scored because they were specific to the intervention study ( Table 3). Furthermore, the majority of the included studies did not contain adequate information to understand the role of potential confounders in their relationship with HRV.

**Table 3.** Quality Index Scoring of included articles.

Study	Reporting	External validity	Bias	Confounding	Power	Total
**Oliveira C *et al***: Journal of Obesity. 2020	7	2	4	3	1	17/32
**De Liao C *et al***: PLOS ONE. 2017	7	2	4	3	1	17/32
**Pope ZC *et al*:** Medicine & science in sports & exercise. 2020	9	3	5	4	2	23/32
**Tonello L *et al*:** Frontiers in physiology. 2016	7	2	4	3	0	16/32
**Kaikkonen KM *et al***: Journal of Physical Activity and Health. 2014	7	2	4	3	1	17/32
**Rennie KL *et al***: American Journal of Epidemiology. 2003	9	3	5	4	2	23/32
**Kiviniemi AM *et al***: Medicine & science in sports & exercise. 2017	9	3	5	4	2	23/32
**Föhr T *et al*:** BMC public health. 2016	7	2	4	3	2	18/32
**Buchheit M, *et al***. Medicine and science in sports and exercise. 2005	7	2	4	3	1	17/32
**May R *et al***:. Journal of physiological anthropology. 2017	7	2	4	3	1	17/32
**Soares-Miranda L *et al*.** Circulation. 2014	9	3	5	4	2	23/32
**Kluttig A *et al***. BMC cardiovascular disorders. 2010	9	3	5	4	2	23/32

### Participant characteristics

The participants were aged between 18 and 83 years and all studies included both men and women, except Tonello *et al.* ^37^ who included young non-menopausal women with higherweight but in good health. Three of the included articles focused on only people with obesity, ^23^^,^^35^^,^^38^ one study had recruited only people with higher weight (Mean BMI = 26.3 ± 4.1 kg/m ^2^) ^37^, and another included people with higher weight and obesity ^36^. In the remaining six studies, it was unclear if participants were with higher weight or obesity, but their mean BMI was >25 kg/m ^2^ (40-45 kg/m ^2^), and one study included people with a BMI both less than and more than 25 kg/m ^2^. ^39^ Physical activity status of the participants is depicted in Table 4.

**Table 4.** Physical activity status of the participants.

Study	Participants	Measurement technique	Physical activity (mean±SD)
**1**. **Oliveira *et al***. Journal of Obesity. 2020	Patients with obesity grade II	Accelerometer	MVPA, ≥150 (min/week) - **98.92 ± 41.00**
**2. De Liao *et al***. PLOS ONE. 2017	Older adults with obesity	GCPS score	Global physical capability score reporting
**3. Pope *et al***. Medicine & science in sports & exercise. 2020	The majority of the participants were with higher weight and obesity - 1157 Underweight - 20 Normal weight - 491	Accelerometer	Vigorous PA (min·d−1) **2.7 ± 6.2** Moderate PA (min·d−1) **33.0 ± 22.0**
**4. Tonello *et al***. Frontiers in physiology. 2016	Young, non-menopausal, with higher weight	Accelerometer	MPA (min/day) **57.22 (18.23)** VPA (min/day) **1.16 (0.93)**
**5. Kaikkonen *et al*.** Journal of Physical Activity and Health. 2014	Adult with obesity	Questionnaire	Reported as frequency (Self-rated physical fitness level good, n (%))
**6. Rennie *et al***. American Journal of Epidemiology. 2003	Participant BMI included above and below 25	Questionnaire	Total physical activity quartile reporting
**7. Kiviniemi *et al***. Medicine & science in sports & exercise. 2017	1,383 men and 1,761 women without cardiorespiratory diseases and diabetes	wrist-worn Polar Active device	MVPA, (min/day) – **Men- 76 (56–99)** **Women- 60 (43–80)**
**8. Föhr *et al***. BMC public health. 2016	Men and women with BMI: 18.5–40.0 kg/m2	HRV-based assessment of PA	Physical activity (mins/week) **186 ± 227**
**9. Buchheit *et al***. Medicine and science in sports and exercise. 2005	Middle-aged adults	Accelerometer	Values are means **±** SEM for weekly time (h_wk_1) spent at specific intensities of physical activity for sedentary (SED), active (ACT), and sportive (SP) subjects: **Moderate activities (4.5–5.9 METs)-**SED- 0.6 **±**0.2 ACT- 0.5 **±**0.1 SPORTIVE- 1.4 **±**0.2 **High-intensity activities (6–8.4 METs)-** SED-0.1 **±** 0.1 ACT-0.1 **±**0.1 SPORTIVE-0.6 **±**0.1
**10. May *et al***. Journal of physiological anthropology. 2017	115 students with BMI: mean (SD): 25.4 (0.5)	Questionnaire	Vigorous activity (minutes per week): **298.5 ± 276.1** Moderate activity (minutes per week): **241.3 ± 298.7**
**11. Soares-Miranda *et al*.** Circulation. 2014	5,201 ambulatories, non-institutionalized men and women	Questionnaire	The median level of leisure-time activity was 630 (158, 1485) kcal/wk.
**12. Kluttig *et al***. BMC cardiovascular disorders. 2010	1,671 participants BMI: Male: 28.5 ± 4.1 kg/m ^2^, Female: 28.5 ± 5.4 kg/m ^2^	Questionnaire	MET - Men - 13.1 ± 10.4 hours per week Women - 9.6 ± 7.6 hours per week

**Figure 2.**
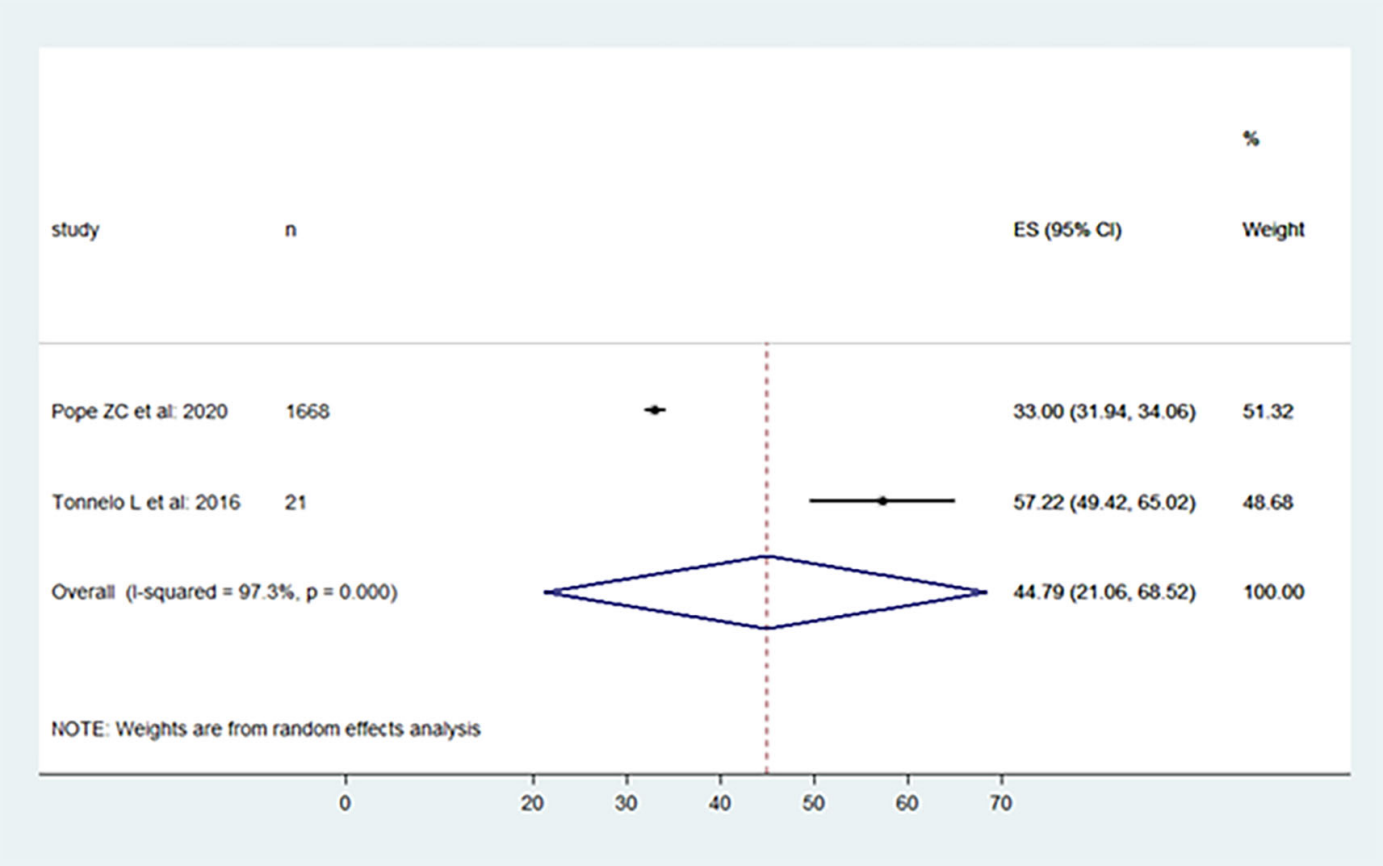
Moderate physical activity level per day.

**Figure 3.**
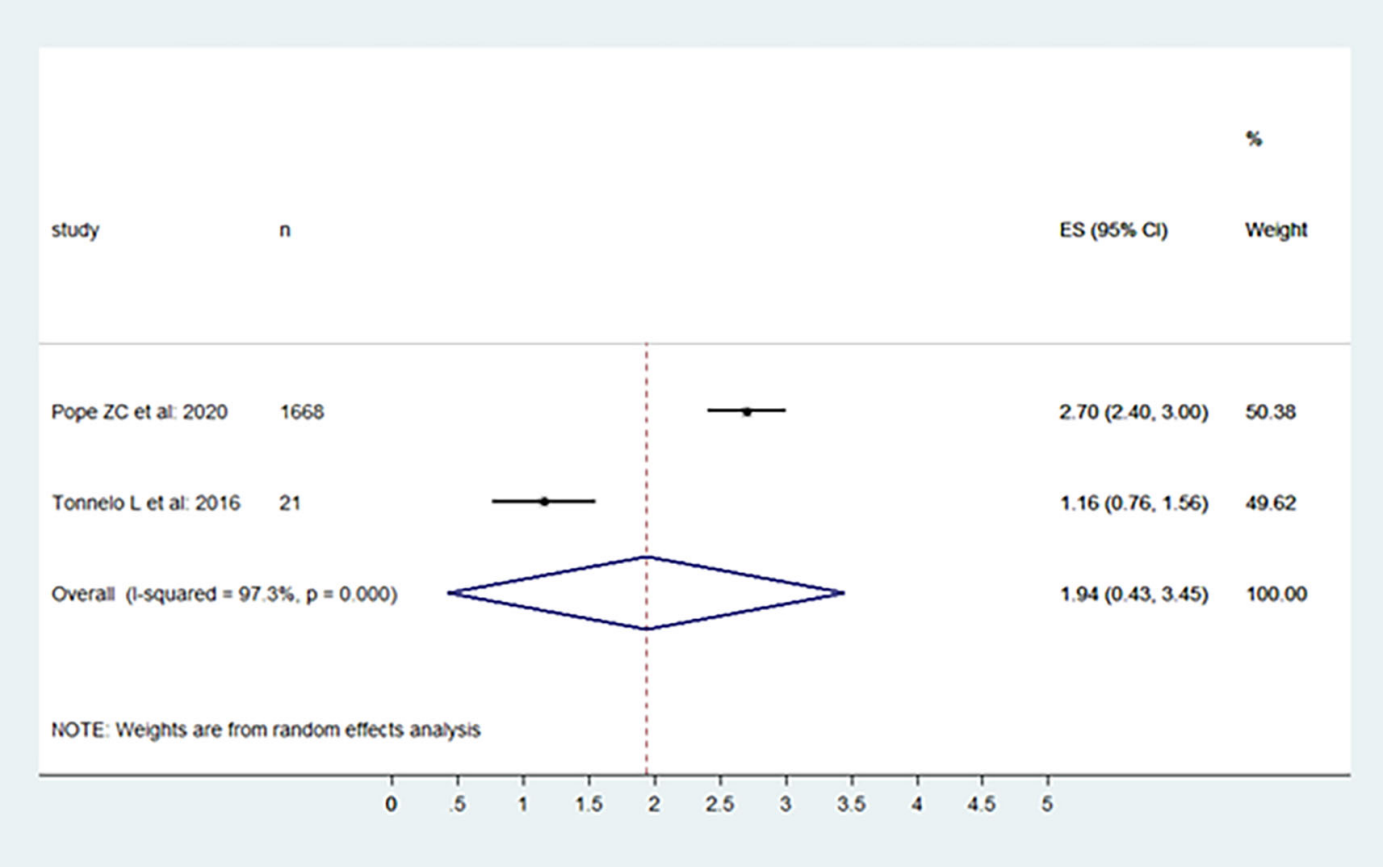
Vigorous physical activity level per day.

**Figure 4.**
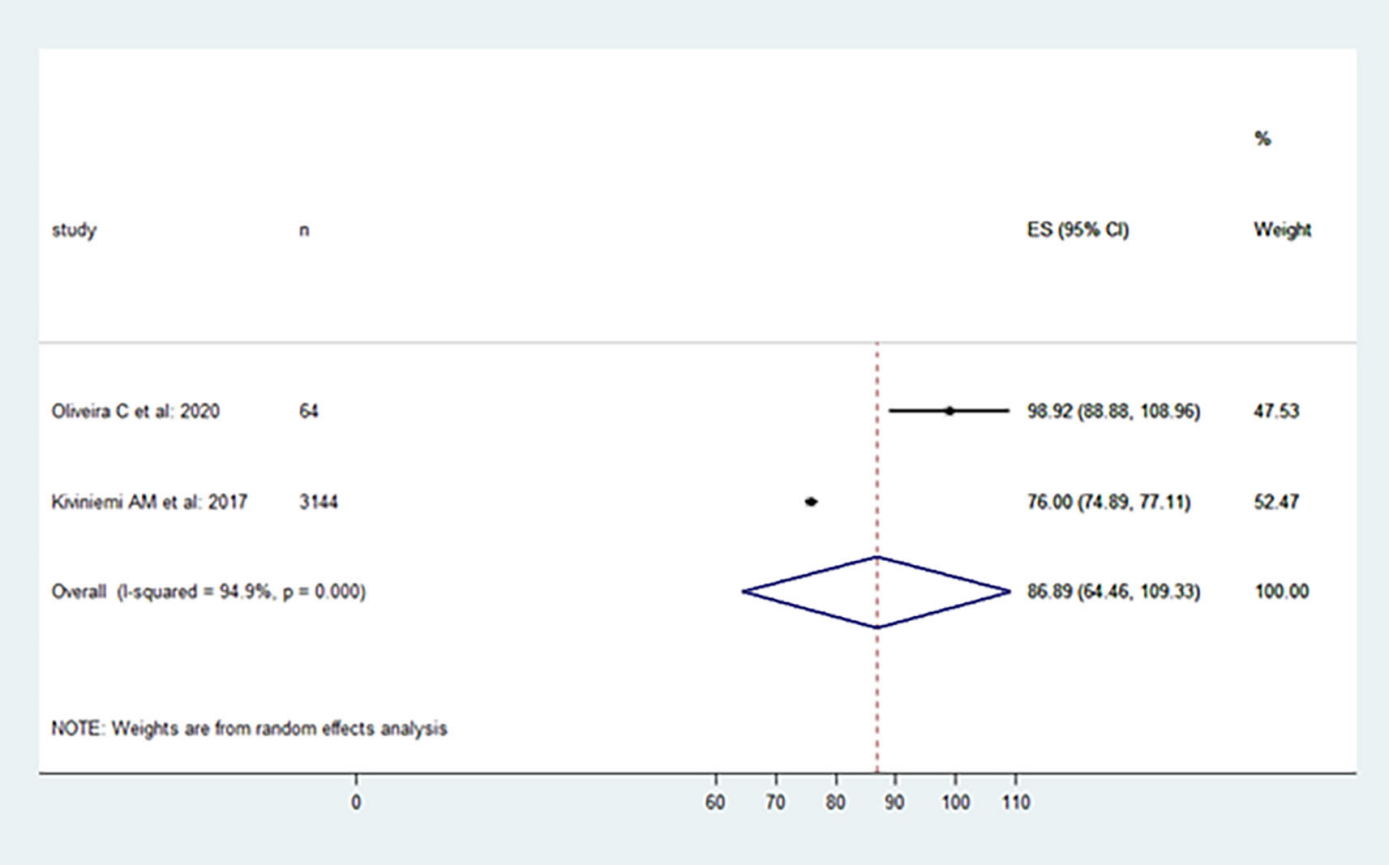
Moderate to vigorous physical activity level per day.

The included studies' levels of physical activity were presented in the following, moderate, vigorous, and moderate to vigorous. Figures 2, 3, and 4 provide specifics of the meta-analysis.

### HRV and physical activity analysis

A brief overview of the studies assessing relationships between HRV and physical activity is presented in Table 5.

**Table 5.** Correlation between heart rate variability variables and physical activity.

Study	Correlation b/w HRV variables and physical activity
**1**. **Oliveira *et al***. Journal of Obesity. 2020	Negative association with HF ( *p* = 0.049) and LF/HF ( *p* = 0 *.*036) and a positive association with LF ( *p* = 0 *.*014) **Table:** Linear regression: Variable **HF (nu)** **LF (nu)** **LF/HF** *β* 95% CI *p* value *β* 95% CI *p* value *β* 95% CI *p* value MVPA (min/day) −0.844 −1.898–0.210 0.114 −1.389 −2.726–−0.051 0.042 0.005 −0.005–0.014 0.303
**2. De Liao *et al.*** PLOS ONE. 2017	The GPCS in old men independently explained an additional 29.9% [ *F* (1, 225) = 232.58, *P <* 0.001], 2.5% [ *F* (1, 225) = 7.95, *P <* 0.01], and 10.6% [ *F* (1, 225) = 39.43, *P <* 0.001] of the variance of ln SDNN, ln rMSSD, and ln HF, respectively; similar results were observed in old women.
**3. Pope *et al.*** Medicine & science in sports & exercise. 2020	Multivariable linear regression **Table:** **SDNN** **rMSSD** MPA 0.027 (−0.011 to 0.064) 0.030 (−0.007 to 0.068) VPA 0.063 (0.028 to 0.099) 0.082 (0.046 to 0.118)
**4. Tonello *et al.*** Frontiers in physiology. 2016	Correlations were observed between VPA and: RMSSD ( *r* = −0.449, *p* = 0.041), HF( *r* = −0.520, *p* = 0.016),
**5.Kaikkonen *et al.*** Journal of Physical Activity and Health. 2014	**Table:** SDNN, ms: Model *R*2 = .403, *P* < .001a Lifetime activity level (per 1 category increase) Regression coefficient 15.359 (3.852 to 26.866) 0.009
**6. Rennie *et al*.** American Journal of Epidemiology. 2003	Dose response relation between greater reported participation in vigorous activity and higher SDNN, low frequency power, and high-frequency power ( *p* < 0.05, *p* < 0.01, and *p* < 0.01, respectively) in men.
**7.Kiviniemi *et al*** *.* Medicine & science in sports & exercise. 2017	rMSSD and MVPA correlation coefficient- r=0.08(p<0.001)
**8. Föhr *et al.*** BMC public health. 2016	**Table:** Results of the linear models Men Women Parameter Estimate 95 % Cl Lower Upper P value Parameter Estimate 95 % Cl Lower Upper P value Stress (%), 24 h Medium physical activity class −1.1405 −2.2144 −0.0667 0.04 −1.9539 −2.7608 −1.1470 <0.001 High physical activity class −3.9695 −5.0062 −2.9328 <0.001 −4.3772 −5.2620 −3.4923 <0.001
**9. Buchheit *et al*** *.* Medicine and science in sports and exercise. 2005	Sportive individuals spent more time in moderate to very high activities than ACT (2.1 ± 0.1 vs 0.6 ± 0.1 h_wk_1; *P* <0.05)
**10. May *et al*** *.* Journal of physiological anthropology. 2017	**Table:** Multiple regression analyses rMSSD R ^2^ (0.10) R ^2^ (0.18) LF R ^2^ (0.07) R ^2^ (0.15) HF R ^2^ (0.08) R ^2^ (0.14) Vigorous activity (minutes) 0.34 (.002) 0.24 (0.04) 0.27 (0.02) .19 (0.10) 0.30 (0.01) 0.20 (0.08) Moderate activity (minutes) 0.04 (0.74) 0.11 (0.31) −0.22 (0.05) −.19 (0.10) 0.06 (0.61) 0.13 (0.25)
**11. Soares-Miranda *et al.*** Circulation. 2014	Multivariable adjustment, leisure-time activity was cross-sectionally related to specific indices including higher SDNN (p-trend=0.001) and higher ULF (p-trend<0.0001)
**12. Kluttig *et al.*** BMC cardiovascular disorders. 2010	Weak and inconsistent associations of higher physical activity with higher time and frequency domain HRV in both sexes

Two studies had reported a negative association between moderate to vigorous physical activity and rMSSD. ^23^^,^^40^ One of these also described a negative correlation between sedentary time and HF (p = 0.049) and LF/HF (p = 0.036), as well as a positive association between sedentary time and LF (p = 0.014). ^23^ Likewise, Föhr *et al.* ^41^ observed that both moderate and vigorous physical activity is linked to rMSSD and the LF/HF ratio.

The GCPS, a method to assess physical activity, was reported to be moderately correlated with ln SDNN and ln HF, with correlation coefficients values of 0.61 (p = 0.001) and 0.59 (p = 0.001), respectively. ^35^ In other three studies, significant correlations were observed between vigorous physical activity (VPA) and rMSSD. ^36^^,^^37^^,^^43^ VPA is associated with SDNN: β _std_= 0.06 [0.03, 0.10]; and rMSSD: β _std_ = 0.08 [0.05, 0.12] according to Pope *et al.* ^36^ and light physical activity (LPA) is associated with only rMSSD (β _std_ = 0.05 [0.01, 0.08]. Tonello *et al.* ^37^ also found correlations between VPA and rMSSD (r = 0.449, p = 0.041), and VPA and HF (r = 0.520, p = 0.016) in their analysis.

One of the studies reported a dose-response relationship between vigorous exercise and higher SDNN, LF power, and HF power (p = 0.05, p = 0.01, and p = 0.01, respectively). ^39^ Leisure-time behavior was cross-sectionally linked to higher SDNN (p _trend_=0.001) and higher ULF (p _trend_<0.0001) according to a study by Soares-Miranda *et al.* ^44^ On the other hand, Kluttig *et al.* found no consistent or statistically significant connection between physical activity and HRV variables. ^45^

## Discussion

This systematic review elucidated the link between physical activity and HRV-derived cardiac autonomic function. We found that rMSSD is negatively correlated to moderate to vigorous physical activity, and sedentary time has a positive relationship with LF. A dose-response relationship between vigorous exercise and frequency-domain HRV measures has been documented (LF and HF). Although the evidence for the effect of age, sex, and diet remains inconclusive, all studies measured physical activity differently and used different equipment to determine HRV. These variables could be obscuring the evidence.

### Physical activity measurement and HRV

The included studies used both objective and subjective methods to measure physical activity. Oliveira *et al.* ^23^, Pope *et al.* ^36^, Tonello *et al.* ^37^, Kiviniemi *et al.* ^40^, and Föhr *et al.* ^41^ all utilized an objective method which included an accelerometer, polar device, or HRV-based physical activity measurement. The remaining studies ^35^^,^^38^^,^^39^^,^^42^^–^^45^ employed questionnaires to assess physical activity. The studies reported that regular physical activity had a positive effect on autonomic control of the heart in adults by decreasing the resting heart rate and an increase in HRV. Further, regular physical activity and aerobic fitness improve a range of health outcomes and lower mortality from all causes. Nevertheless, the use of objective tools to assess physical activity and thorough body composition measurements has been less frequent.

### HRV measurement

The included studies used a variety of equipment to record HRV–V800 Polar, ANS watch monitor, GE MC1200 for resting electrocardiography (ECG); RS800CX Polar heart rate monitor, Polar R-R recorder, 12-lead electrocardiogram, Firstbeat Bodyguard device for ambulatory HRV recording; ECG Holter, BIOPAC MP-36 system, 24-hour Holter and Modular ECG Analysis System. However, the use of different equipment to measure HRV in the included studies limits the scope of comparison of results between the studies. Many confounding factors for HRV were identified which included the level of physical activity, recording time, room temperature, individual stress or nutrition, gender, and several hormones. ^23^^,^^35^^–^^45^ The reporting may also be influenced by the HRV data or log transformation of the data.

### Physical activity and HRV

Oliveira *et al.* ^23^ stated that physical activity and HRV-derived cardiac autonomic activity, rMSSD, have a negative relationship with moderate to VPA and higher levels of MVPA were associated with reduced LF; however, their participants were with severe obesity with only minimal time spent on MVPA (98.92 ± 41.00 min/week). Another important finding was the association between WC and the deterioration of vagal tone. ^23^ In the same article, sedentary time was linked to all of the frequency domain indices, with a negative correlation with the parasympathetic component and sympathovagal HRV balance, and a positive association with cardiac sympathetic modulation. ^23^ This suggests that PA may protect the heart, as opposed to increased sympathetic tone. Higher levels of PA are also associated with a favourable modulation of the cardiac sympathetic nervous system in severely obese individuals. As a result, PA reduces the risk of cardiovascular disease associated with obesity through enhancing autonomic function. ^23^ Similarly, De Liao *et al.* ^32^ reported higher cardiac frequency and a decrease in the HF index in elderly persons with higher weight, and a lower HRV in the poor physical capacity group compared to the high physical capacity group.

On analyzing further, Kiviniemi *et al.* ^40^ found a negative relationship between rMSSD and MVPA where physical activity was monitored continuously over two weeks, which can be considered representative of the overall current physical activity level. However, the authors stated that cardiorespiratory fitness was a better predictor of cardiac autonomic function than MVPA.

Pope *et al.* ^36^ found independent associations between accelerometer-estimated VPA, MPA, and LPA and time-domain HRV measures. Their findings suggested that LPA participation is affordable to a large portion of the population regardless of disease status or age, arguing for more LPA participation to replace sedentary time, highlighting the benefits of LPA. When compared to sedentary subjects, Buchheit *et al.* ^42^ found that those with the greatest self-reported MVPA had greater SDNN and rMSSD values as well as higher HF power. According to Soares L *et al.* ^44^ prospective study, walking and leisure activities are connected to improved HRV. Additionally, older people who increased their walking distance or pace over a five-year period of follow-up had a more favourable HRV than those who decreased it. Kleiger RE *et al.* ^47^ also observed the benefits of physical activity and significant correlations between physical activity and higher SDNN and ULF. ^47^ Likewise, Kluttig *et al.* ^45^ observed that physical activity was inconsistently related with HRV and HRV were closely linked to biomedical risk factors, resulting in a high predictive capacity for potential cardiovascular events like diabetes and obesity.

## Conclusion

This review found that physical activity and obesity indices were both independently associated with changes in the cardiac autonomic modulation of obese individuals. Improved cardiac autonomic function has been shown to have positive effects on health outcome, it is crucial to promote it because PA greatly impacts adipocity indices. In this systematic review, we observed a wide range of responses to physical activity and HRV (time and frequency domain variables); however, the existing literature contains a variety of methodologies and equipment for quantifying physical activity and measuring HRV. Furthermore, the evidence for a definite association between different levels of physical activity and HRV is insufficient. Future studies should aim to delve deeper into the influence of intensity and type of physical activity on cardiovascular parameters accounting for all potential regulated confounders. Also, the variability in the equipment used to determine HRV makes it difficult to draw objective conclusions, thereby limiting generalizability.

## Ethical approval

Formal ethical approval is not required as this article is a review paper.

## Data Availability

All of the data that underlies the results are included in the article and no additional source data are required. Figshare: PRISMA_2020_checklist for ‘Association of physical activity and heart rate variability in people with overweight and obesity A systematic review’. https://doi.org/10.6084/m9.figshare.21119554.v1. ^48^ Data are available under the terms of the Creative Commons Attribution 4.0 International license (CC-BY 4.0).
