# Supplementary material for: Association of physical activity and heart rate variability in people with overweight and obesity: A systematic review
Source: F1000Res. 2023 Feb 10;12:156. [Version 1] doi: 10.12688/f1000research.124707.1 (PMC9982191; doi:10.12688/f1000research.124707.1)
Supplement: Peer review history

# Peer review: Reviewer response for version 1

**DOI:** 10.5256/f1000research.136931.r163548

**Published:** 2023-03-02

**Authors:** Aishwarya Nair

**Affiliations:** 1 Nitte Institute of Physiotherapy, NITTE (Deemed to be University), Mangaluru, Karnataka, India

The article has been well drafted with a thorough literature search. 

**General:**

1. The grammar of the overall manuscript can be upgraded especially the Introduction.

**Abstract:**

1. Background for overweight can be mentioned in the abstract along with the distinction between obesity and overweight since these words are mentioned in the title.
2. In a few places, the terms higher weight/overweight/obesity have been used interchangeably. This has been observed throughout the manuscript. A uniformity can be incorporated for the same.

**Introduction:**

- No comments.

**Methods:**

- No comments.

**Discussion:**

1. In understanding the association between heart rate variability and physical activity, it is advised to add more points in the discussion that give an understanding regarding the mechanism behind it.

**Conclusion:**

1. The impact of PA on obesity and overweight can be elaborated as a summary.
2. Limitations and future scope can be mentioned in terms of obesity, overweight, PA as well in addition to HRV and PA.

Are the rationale for, and objectives of, the Systematic Review clearly stated?

Yes

Is the statistical analysis and its interpretation appropriate?

Yes

Are sufficient details of the methods and analysis provided to allow replication by others?

Yes

Are the conclusions drawn adequately supported by the results presented in the review?

Yes

Reviewer Expertise:

Rehabilitation, Physical Fitness, Cardiac performance, Exercise Physiology, Physical acitivity

I confirm that I have read this submission and believe that I have an appropriate level of expertise to confirm that it is of an acceptable scientific standard.

# Peer review: Reviewer response for version 1

**DOI:** 10.5256/f1000research.136931.r163550

**Published:** 2023-02-28

**Authors:** Rajat M. Singh

**Affiliations:** 1 Physiotherapy, Ravi Nair Physiotherapy College, Datta Meghe Institute of Medical Sciences, Wardha, Maharashtra, India, Sawangi, Maharashtra, India

This topic is interesting, and the manuscript is well-written. The abstract, methodology, results, discussion, and conclusion are all well-written sections of the manuscript. The overall quality of the writing makes the manuscript acceptable. Few suggestions from my end:

1. **Discussion section** - **HRV Measurement**: Describe the HRV measurement in detail and discuss its usefulness.
2. **Discussion section - Physical activity and HRV**: ****Discuss physical activity and RMSSD, PNN50, and physical activity and LF, HF, and LF/HF ratio.
3. Provide details of the confounding measures if it is reported in the included studies.

Are the rationale for, and objectives of, the Systematic Review clearly stated?

Yes

Is the statistical analysis and its interpretation appropriate?

Yes

Are sufficient details of the methods and analysis provided to allow replication by others?

Yes

Are the conclusions drawn adequately supported by the results presented in the review?

Yes

Reviewer Expertise:

Physical activity,  Obesity and Health promotion fitness

I confirm that I have read this submission and believe that I have an appropriate level of expertise to confirm that it is of an acceptable scientific standard.

# Peer review: Reviewer response for version 1

**DOI:** 10.5256/f1000research.136931.r163549

**Published:** 2023-02-24

**Authors:** Jaya Shanker Tedla

**Affiliations:** 1 Department of Medical Rehabilitation Sciences, College of Applied Medical Sciences, King Khalid University, Abha, Saudi Arabia

The article is well documented. The authors have done an excellent job of accumulating evidence for their hypothesis. Overweight and obesity are burning common problems throughout the world, and physical activity has a very high impact on this. The manuscript follows proper technique and is well presented. It is an interesting article. These kinds of articles are important in the current situation. This paper can be accepted with a few minor suggestions which need to be addressed.

1. The current obesity recommendation for physical activity should be highlighted in the introduction section. Regular physical activity has also been shown to help control and prevent non-communicable diseases.
2. Despite the many confounders, HRV is a promising marker of cardiac autonomic function. If those confounders like diet and sleep were taken into account in the included study, the authors need to mention them in the discussion section.

Are the rationale for, and objectives of, the Systematic Review clearly stated?

Yes

Is the statistical analysis and its interpretation appropriate?

Yes

Are sufficient details of the methods and analysis provided to allow replication by others?

Yes

Are the conclusions drawn adequately supported by the results presented in the review?

Yes

Reviewer Expertise:

Physical Therapy, Rehabilitation, Neurology, Pediatrics

I confirm that I have read this submission and believe that I have an appropriate level of expertise to confirm that it is of an acceptable scientific standard, however I have significant reservations, as outlined above.
